# Congenital Deficiency of Conventional Dendritic Cells Promotes the Development of Atopic Dermatitis-Like Inflammation

**DOI:** 10.3389/fimmu.2021.712676

**Published:** 2021-07-27

**Authors:** Yotaro Nishikawa, Tomohiro Fukaya, Takehito Fukui, Tomofumi Uto, Hideaki Takagi, Junta Nasu, Noriaki Miyanaga, Dieter Riethmacher, Narantsog Choijookhuu, Yoshitaka Hishikawa, Masahiro Amano, Katsuaki Sato

**Affiliations:** ^1^Division of Immunology, Department of Infectious Diseases, Faculty of Medicine, University of Miyazaki, Miyazaki, Japan; ^2^Department of Dermatology, Faculty of Medicine, University of Miyazaki, Miyazaki, Japan; ^3^Japan Agency for Medical Research and Development (AMED), Tokyo, Japan; ^4^Department of Oral and Maxillofacial Surgery, Faculty of Medicine, University of Miyazaki, Miyazaki, Japan; ^5^Department of Otolaryngology, Head and Neck Surgery, Faculty of Medicine, University of Miyazaki, Miyazaki, Japan; ^6^Department of Biomedical Sciences, School of Medicine, Nazarbayev University, Nur-Sultan, Kazakhstan; ^7^Division of Histochemistry and Cell Biology, Department of Anatomy, Faculty of Medicine, University of Miyazaki, Miyazaki, Japan

**Keywords:** atopic dermatitis, dendritic cells, type 2 immune responses, immune homeostasis, homeostatic feedback loop

## Abstract

Atopic dermatitis (AD) is a common pruritic inflammatory skin disease characterized by impaired epidermal barrier function and dysregulation of Thelper-2 (T_H_2)-biased immune responses. While the lineage of conventional dendritic cells (cDCs) are implicated to play decisive roles in T-cell immune responses, their requirement for the development of AD remains elusive. Here, we describe the impact of the constitutive loss of cDCs on the progression of AD-like inflammation by using binary transgenic (Tg) mice that constitutively lacked CD11c^hi^ cDCs. Unexpectedly, the congenital deficiency of cDCs not only exacerbates the pathogenesis of AD-like inflammation but also elicits immune abnormalities with the increased composition and function of granulocytes and group 2 innate lymphoid cells (ILC2) as well as B cells possibly mediated through the breakdown of the Fms-related tyrosine kinase 3 ligand (Flt3L)-mediated homeostatic feedback loop. Furthermore, the constitutive loss of cDCs accelerates skin colonization of *Staphylococcus aureus (S. aureus)*, that associated with disease flare. Thus, cDCs maintains immune homeostasis to prevent the occurrence of immune abnormalities to maintain the functional skin barrier for mitigating AD flare.

## Introduction

Dendritic cells (DCs) are important professional antigen (Ag)-presenting cells (APCs) that play pleiotropic roles in integration and fine-tuning of the immune system, bridging between innate and adaptive immunity (1–3). DCs encompass the functionally distinguishable two principal lineages, classical or conventional DCs (cDCs) and plasmacytoid DCs (pDCs) (1–3). cDCs constitute unique APCs endowed with the unrivaled immunogenicity with remarkable expressions of major histocompatibility complex (MHC) and costimulatory molecules that license them for priming naïve T cells to differentiate into various types of effector T (T_eff_) cells (1–3). On the other hand, pDCs are characterized to secrete a large amount of type-I interferon (IFN) following recognition of viral nucleic acids through endosomal toll-like receptor (TLR)7/9 for the initiation of antiviral responses (3, 4). Conversely, DCs are also implicated to be crucial for generating immune tolerance, and that mechanism includes immune suppression by CD4^+^Foxp3^+^ regulatory T (T_reg_) cells under the homeostatic conditions and certain environmental conditions (3, 5–9).

Atopic dermatitis (AD) is a chronic relapsing inflammatory skin disorder associated with itchy eczematous skin lesion (10–12). The pathophysiology of AD is complex and results from impaired epidermal barrier function and cutaneous inflammation as well as a type 2 helper T (T_H_2)-skewed immune dysregulation with elevated serum immunoglobulin (Ig)E level and peripheral blood eosinophilia, caused by the interaction between genetic and environmental predispositions (10–15). Moreover, the pruritic inflammatory lesion of AD increases the susceptibility to microbial colonization such as infections to *Staphylococcus aureus* (*S. aureus*) that associated with disease flare (11, 12, 16–18). While the lineage of DCs have been considered to play decisive roles in the pathogenesis of cutaneous allergic diseases (19–29), the intrinsic role of cDCs in the maintenance of skin immune homeostasis in the steady-state conditions that impacts the onset of eczematous inflammation remains unclear.

In this study, we address how the congenital deficiency of CD11c^hi^ cDCs affects the development of experimental pruritic dermatitis by using binary transgenic (Tg) mice that constitutively lacked CD11c^hi^ cDCs (30). Unexpectedly, the constitutive loss of cDCs caused the immune dysregulation leading to the exacerbation of AD-like inflammation, which demonstrates the first time that cDCs mediate the maintenance of skin immune homeostasis mitigating the allergic skin disorders.

## Materials and Methods

### Mice

The following 6- to 12-week-old mice were used in this study. C57BL/6 mice (Japan Clea), B6.Cg-Tg(Itgax-cre)1-1Reiz/J mice (31) (CD11c-Cre mice; The Jackson Laboratory), and R26:lacZbpA^flox^DTA mice (R-DTA mice) (32). R-DTA mice and CD11c-Cre mice, which had been backcrossed for ten generations on to C57BL/6 mice, were cross-mated for generating CD11c-Cre:R-DTA mice used as ΔCD11c^hi^ cDC mice, and their WT littermates were used as CD11c^hi^ cDC-sufficient control mice. B6.CD45.1^+^OT-I TCR Tg mice harboring OVA-specific CD8^+^ T cells (B6.CD45.1^+^OT-I mice) and B6.CD45.1^+^OT-II TCR Tg mice harboring OVA-specific CD4^+^ T cells (B6.CD45.1^+^OT-II mice) were generated as described previously (4, 33–36). All mice were bred and maintained in specific pathogen-free conditions in the animal facility at the University of Miyazaki. All experiments were performed in accordance with institutional guidelines and approved by the Animal Experiment Committee and Gene Recombination Experiment Committee at the University of Miyazaki.

### Additional Methods

Tissue and cell isolation (4, 5, 9, 33–37), flow cytometry (4, 5, 9, 33–37), quantitative reverse transcription polymerase chain reaction (RT-PCR) (9, 36), measurement of serum Ig, Fms-related tyrosine kinase 3 ligand (Flt3L), and cytokines, AD-like inflammation (9, 13, 15, 37–39. 15), adoptive transfer (4, 33–36), histopathologic assessment (9, 37), immunohistochemical analysis, and bacterial culture (16) are described in **Supplementary Material**.

### Statistical Analysis

Data are expressed as the mean ± s.d from three to ten individual samples in a single experiment, and we performed at least three independent experiments. The statistical significance of the differences between the values obtained was evaluated by two-sided paired student t-test or two-way ANOVA. A P value of <.05 was considered significant.

## Results

### Constitutive Loss of cDCs Alters the Immune Cell Composition Under Steady-State Conditions

To address the role of cDCs in the progression of AD-like inflammation, we generated a mouse model that constitutively lacks CD11c^hi^ cDCs (30) by crossing CD11c-Cre bacterial artificial chromosome (BAC) Tg mice (CD11c-Cre mice) (31) to mice that harbor the diphtheria toxin (DT) α chain (DTA) under control of a loxP-flanked stop cassette in the ubiquitously expressed ROSA26 locus (R-DTA mice) (32) to produce CD11c-Cre:R-DTA double-Tg mice, referred to as CD11c-Cre:R-DTA mice. Although similar absolute cell numbers of leukocytes were observed in spleen (Spl) and ear-draining lymph nodes (EDLNs) between wild-type (WT) littermates and CD11c-Cre:R-DTA mice (**Figures S1A, B **in** Supplementary Materials**), CD11c-Cre:R-DTA mice revealed almost complete elimination of CD11c^hi^ cDCs in Spl and EDLNs in the homeostatic conditions when compared with WT mice (**Figures S1C, D**, **S2A **in** Supplementary Materials**). Histological analysis confirmed the lack of CD11c^hi^ cDCs in Spl of CD11c-Cre:R-DTA mice (**Figure S2B **in** Supplementary Materials**). Furthermore, normal composition of pDCs was observed in Spl of CD11c-Cre:R-DTA mice, while their proportion were slightly reduced in EDLNs (**Figures S1C, D **in** Supplementary Materials**). On the other hand, epidermal LCs highly expressing CD11c were barely detected in ear epidermis of CD11c-Cre:R-DTA mice (**Figures S2C–E **in** Supplementary Materials**), that were differently observed in the previous report (30).

Collectively, CD11c-Cre:R-DTA mice shows the selective constitutive ablation of CD11c^hi^ cDC subsets (called “ΔCD11c^hi^ cDC mice” hereafter).

We also examined the influence of the constitutive elimination of CD11c^hi^ cDCs on the composition of leukocytes in lymphoid and peripheral tissues under steady-state condition. ΔCD11c^hi^ cDC mice displayed the reduced proportions of CD4^+^ T cells, CD8^+^ T cells, γδT cells, CD4^+^ST2^+^ cells known as pathogenic T_H_2 cells (36), CD4^+^Foxp3^+^ T_reg_ cells, and natural killer T (NKT) cells, whereas they exhibited the enhanced proportions of neutrophils, eosinophils, and basophils/mast cells (MCs) in Spl as compared with WT mice (**Figure S1C **in** Supplementary Materials**). Similar results were observed in the proportions of CD4^+^ T cells, CD8^+^ T cells, CD4^+^Foxp3^+^ T_reg_ cells, neutrophils, and eosinophils in EDLNs between WT mice and ΔCD11c^hi^ cDC mice (**Figure S1D **in** Supplementary Materials**).

Taken together, these results indicate that the constitutive absence of CD11c^hi^ cDCs leads to abnormal composition of leukocytes in lymphoid tissues.

### Constitutive Loss of cDCs Causes the Spontaneous Inflammatory Responses

We compared the inflammatory status between WT mice and ΔCD11c^hi^ cDC mice in the homeostatic conditions. ΔCD11c^hi^ cDC mice exhibited the higher serum productions of tumor necrosis factor (TNF)-α, IFN-γ, interleukin (IL)-4, IL-5, IL-13, IL-17A, and Flt3L than WT mice (**Figure S3A **in** Supplementary Materials**). When compared with WT mice, ΔCD11c^hi^ cDC mice exhibited the enhanced expressions of *Il4*, *Il13*, *Il33*, *Tarc*, and *S100a8* known as alarmin, whereas they showed the reduced expressions of *Saa2* and *Loricrin* known as the barrier-related molecules in ear cutaneous tissues (**Figure S3B **in** Supplementary Materials**).

Taken together, these results indicate that the constitutive depletion of CD11c^hi^ cDCs triggers spontaneous inflammation in periphery and cutaneous tissues.

### Constitutive Loss of cDCs Causes the Adaptive Immune Abnormality Under Steady-State Conditions

To determine the role of CD11c^hi^ cDCs in the initiation of Ag-specific T-cell responses, we adaptively transferred eFluor670-labelled OT-II^+^CD4^+^ T cells or OT-I^+^CD8^+^ T cells expressing the ovalbumin (OVA)-specific T-cell receptor (TCR) (4, 33–36) into WT mice or ΔCD11c^hi^ cDC mice, administrated OVA protein, and monitored their Ag-specific division in Spl and EDLNs. In contrast to the marked division of OT-II^+^CD4^+^ T cells or OT-I^+^CD8^+^ T cells in Spl and EDLNs in WT mice following systemic administration of OVA protein, their responses severely diminished in ΔCD11c^hi^ cDC mice (**Figure S4 **in** Supplementary Materials** **)**.

Collectively, these results indicate that the constitutive deficiency of CD11c^hi^ cDCs abolishes the Ag-specific priming of T cells *in vivo*.

We also addressed the influence of the constitutive deficiency of CD11c^hi^ cDCs on the emergences of CD4^+^ T_eff_ cells and innate lymphoid cells (ILCs) under steady state conditions. In Spl, ΔCD11c^hi^ cDC mice showed lower proportions of CD4^+^IFN-γ^+^ T_H_1 cells, CD4^+^IL-5^+^ T_H_2 cells, and CD4^+^IL-13^+^ T_H_2 cells than WT mice (**Figure S5A **in** Supplementary Materials**). Conversely, ΔCD11c^hi^ cDC mice displayed higher frequencies of Lin^-^Thy1.2^+^GATA3^+^ ILC2, Lin^-^Thy1.2^+^GATA3^+^IL-5^+^ ILC2, Lin^-^Thy1.2^+^GATA3^+^IL-13^+^ ILC2, Lin^-^RORγt^+^ ILC3, and Lin^-^RORγt^+^IL-17A^+^ ILC3 in Spl than WT mice (**Figure S5B **in** Supplementary Materials**). On the other hand, ΔCD11c^hi^ cDC mice exhibited higher or lower proportions of Lin^-^Thy1.2^+^GATA3^+^ ILC2 as well as Lin^-^Thy1.2^+^GATA3^+^IL-5^+^ ILC2 and Lin^-^Thy1.2^+^GATA3^+^IL-13^+^ ILC2 or CD4^+^IL-13^+^ T_H_2 cells in EDLNs **(** **Figures S5C, D **in** Supplementary Materials**).

Collectively, these results indicate that the constitutive loss of CD11c^hi^ cDCs enhances the generation of ILC2, while it impairs the initiation of Ag-specific T-cell responses and the generation of CD4^+^ T_eff_ cells.

We further explored the influence of the constitutive ablation of CD11c^hi^ cDCs in B-cell responses in the homeostatic conditions. ΔCD11c^hi^ cDC mice showed more potent serum productions of IgG and IgE than WT mice (**Figure S6A **in** Supplementary Materials**). Furthermore, ΔCD11c^hi^ cDC mice displayed higher proportions of IgM^+^ B cells, $\text{Ig}{\text{G}}_{1}^{+}$ B cells and IgE^+^ B cells in Spl and EDLNs (**Figures S6B, C **in** Supplementary Materials**). Similar results were observed in the frequencies of IgM^+^ B cells, $\text{Ig}{\text{G}}_{1}^{+}$ B cells and IgE^+^ B cells in germinal center (GC) of Spl and EDLNs (**Figures S6D, E **in** Supplementary Materials**). On the other hand, ΔCD11c^hi^ cDC mice showed lower or higher proportion of IgM^+^ plasma cells or IgE^+^ plasma cells in bone marrow (BM) than WT mice (**Figure S6F **in** Supplementary Materials**).

Taken together, these results indicate that the constitutive deficiency of CD11c^hi^ cDCs promotes B-cell responses for the enhanced production of antibody (Ab) in the homeostatic conditions.

### Constitutive Loss of cDCs Exacerbates the Development of AD-Like Inflammation

**Figure 1:**
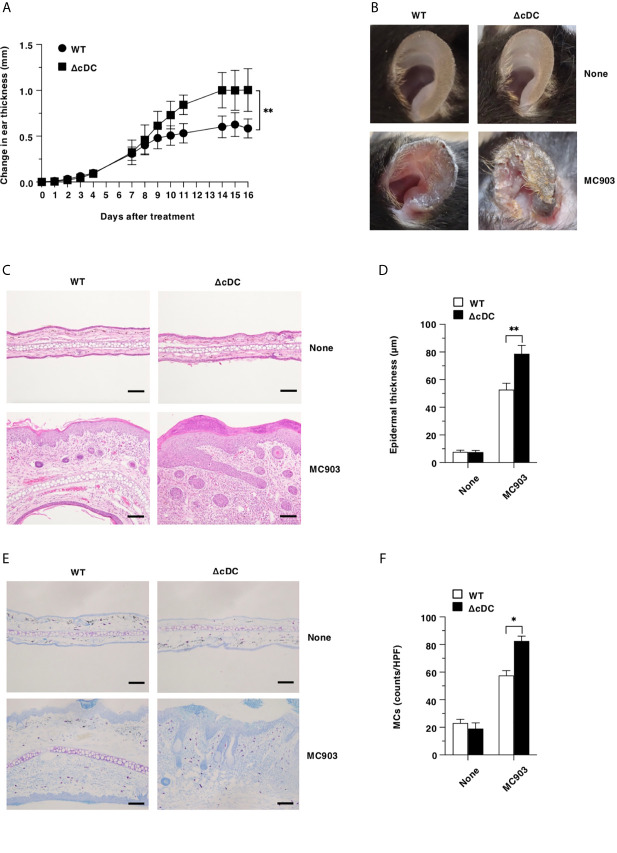
Constitutive loss of cDCs aggravates the development of AD-like inflammation. **(A, B)** WT mice and ΔCD11c^hi^ cDC mice (n = 14 per group) were treated topically with MC903 on the left ear skin. **(A)** Ear thickness was evaluated for 16 days. Data were obtained from fourteen individual samples in a single experiment. **(B)** Representative pictures of ear skin lesions at days 0 (None) and 16 (MC903). **(C, D)** Representative hematoxylin and eosin (H&E) sections (magnification; 200x) of ear skin **(C)**, and epidermal thickness **(D)** was evaluated at days 0 (None) and 16 (MC903). Bars indicate 100 μm. Data were obtained from five individual samples in a single experiment. **(E, F)** Representative toluidine blue sections (magnification; 200x) for detecting MCs of ear skin **(E)**, and the quantification of MCs of the skin **(F)** at days 0 (None) and 16 (MC903). Bars indicate 100 μm. HPF, high performance field. Data were obtained from three individual samples in a single experiment. *P < .05, **P < .01 compared with WT mice by two-sided paired student *t*-test. All data are representative of at least 3 independent experiments.

Lineage of DCs are believed to be required for the initiation and progression of the pathogenesis of cutaneous allergic diseases (19–29). We therefore sought to determine the effect of the constitutive elimination of CD11c^hi^ cDCs on the development of AD-like pathogenesis by using a low calcemic analogue of vitamin D3 known as MC903 (calcipotriol) (13, 15, 15, 38, 39). Unexpectedly, ΔCD11c^hi^ cDC mice exhibited a more prominent AD-like inflammation with significant scaling and thickening than WT mice upon topical application of MC903 on the ear skin (**Figures 1A, B**). Furthermore, histological analyses revealed that ΔCD11c^hi^ cDC mice displayed a more significant epidermal hyperplasia in ear skin as well as cutaneous infiltration of mononuclear cells, including MCs than WT mice following topical application of MC903 (**Figures 1C–F**).

Collectively, these results indicate that the constitutive loss of CD11c^hi^ cDCs aggravates the development of AD-like inflammation.

### Constitutive Loss of cDCs Promotes Type 2 Immune Responses Under Eczematous Inflammatory Conditions

**Figure 2:**
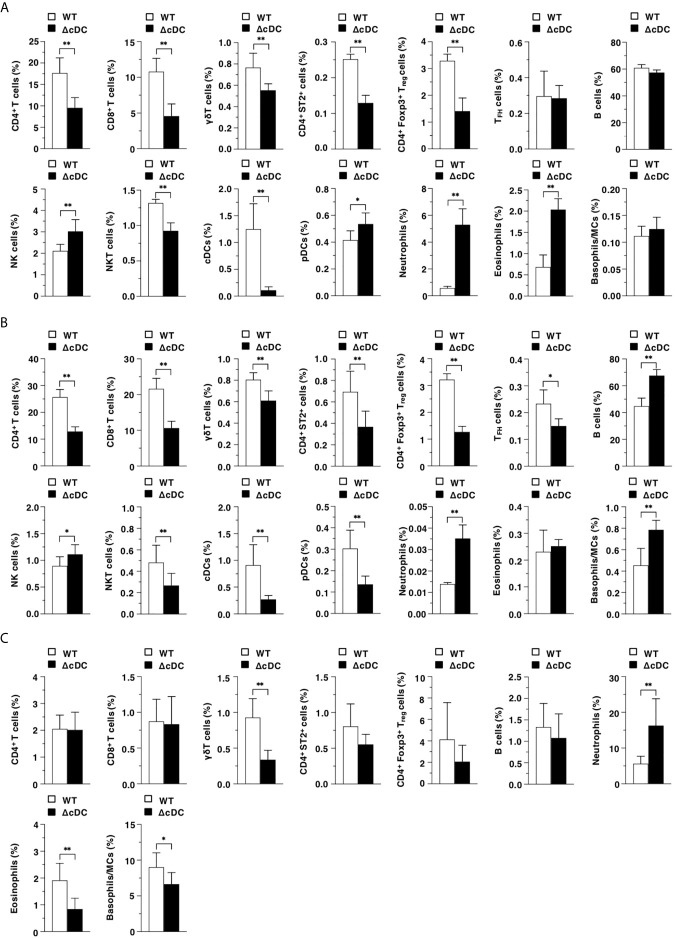
Constitutive loss of cDCs accelerates the AD-associated abnormal immune cell composition. The frequency of leukocytes in Spl **(A)**, EDLNs **(B)**, ear skin **(C)** obtained from WT mice and ΔCD11c^hi^ cDC mice (n = 10 per group) at 16 days after topical application with MC903. Data are obtained from ten individual samples in a single experiment. *P < .05, **P < .01 compared with WT mice by two-sided paired student *t*-test. All data are representative of at least 3 independent experiments.

We compared the constitutions of leukocytes in lymphoid and peripheral tissues between WT mice and ΔCD11c^hi^ cDC mice after topical application of MC903. ΔCD11c^hi^ cDC mice exhibited the reduced frequencies of CD4^+^ T cells, CD8^+^ T cells, γδT cells, CD4^+^ST2^+^ cells, CD4^+^Foxp3^+^ T_reg_ cells, and NKT cells, while they exhibited the enhanced proportions of NK cells, pDCs, neutrophils, and eosinophils in Spl as compared with WT mice (**Figure 2A**). In EDLNs, ΔCD11c^hi^ cDC mice exhibited the reduced frequencies of T cell subsets, NKT cells, and pDCs, whereas they exhibited the enhanced proportions of B cells, NK cells, neutrophils, and basophils/MCs as compared with WT mice (**Figure 2B**). On the other hand, ΔCD11c^hi^ cDC mice exhibited lower or higher frequencies of γδT cells, eosinophils, and basophils/MCs or neutrophils in eczematous ear skin than WT mice (**Figure 2C**).

Taken together, these results indicate that the constitutive absence of CD11c^hi^ cDCs accelerates the AD-associated abnormal composition of leukocytes in lymphoid tissues and eczematous skin lesion.

**Figure 3:**
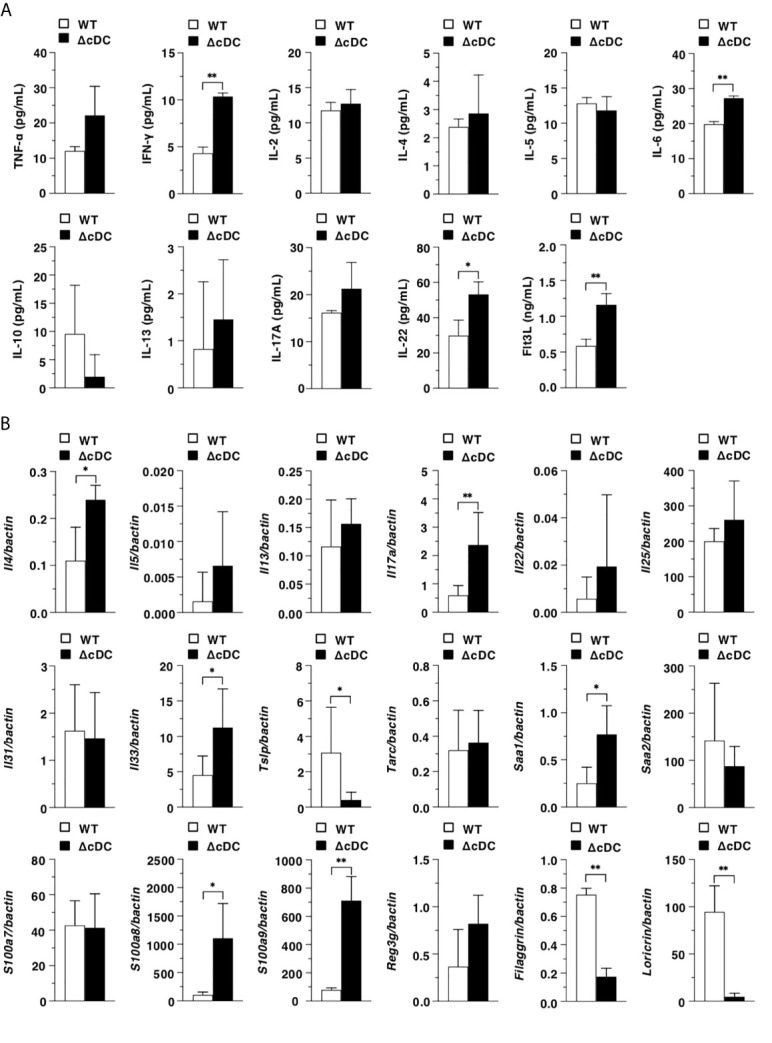
Constitutive loss of cDCs enhances the AD-associated inflammation. **(A)** Serum production of cytokines in WT mice and ΔCD11c^hi^ cDC mice (n = 4 per group) at 16 days after topical application with MC903. Data are obtained from four individual samples in a single experiment. **(B)** Transcriptional expression of inflammation- and epithelium-related molecules in ear skin obtained from WT mice and ΔCD11c^hi^ cDC mice (n = 9 per group) at 16 days after topical application with MC903. Data are obtained from nine individual samples in a single experiment. *P < .05, **P < .01 compared with WT mice by two-sided paired student *t*-test. All data are representative of at least 3 independent experiments.

We next examined the differences in the inflammatory status between WT mice and ΔCD11c^hi^ cDC mice after topical application of MC903. ΔCD11c^hi^ cDC mice showed the higher serum productions of IFN-γ, IL-6, IL-22, and Flt3L than WT mice (**Figure 3A**). In ear eczematous tissues, ΔCD11c^hi^ cDC mice exhibited the higher or lower expressions of *Il4*, *Il17a*, *Il33*, *Saa1*, *S100a8*, and *S100a9*, or *Tslp, Filaggrin,* and *Loricrin* than WT mice **(** **Figure 3B**).

Taken together, these results indicate that the constitutive depletion of CD11c^hi^ cDCs enhances the AD-associated inflammation in periphery and eczematous skin.

**Figure 4:**
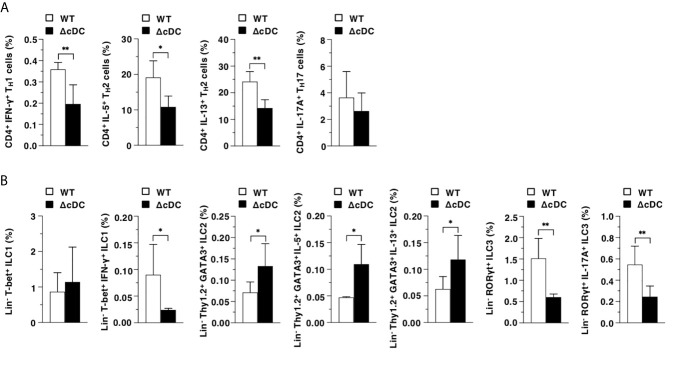
Constitutive loss of cDCs reciprocally controls the composition of CD4^+^ T_eff_ cells and ILCs during onset of AD-like inflammation. The frequency of the subsets of T cells **(A)** and ILCs **(B)** in EDLNs obtained from WT mice and ΔCD11c^hi^ cDC mice (n = 6 per group) at 16 days after topical application with MC903. Data are obtained from six individual samples in a single experiment. *P < .05, **P < .01 compared with WT mice by two-sided paired student *t*-test. All data are representative of at least 3 independent experiments.

We also evaluated the differences in the generation of CD4^+^ T_eff_ cells and ILCs in EDLNs between WT mice and ΔCD11c^hi^ cDC mice after topical treatment of MC903. The proportions of CD4^+^IFN-γ^+^ T_H_1 cells, CD4^+^IL-5^+^ T_H_2 cells, CD4^+^IL-13^+^ T_H_2 cells, Lin^-^T-bet^+^IFN-γ^+^ ILC1, Lin^-^RORγt^+^ ILC3, and Lin^-^RORγt^+^IL-17A^+^ ILC3 were decreased in ΔCD11c^hi^ cDC mice, whereas those of Lin^-^Thy1.2^+^GATA3^+^ ILC2, Lin^-^Thy1.2^+^GATA3^+^IL-5^+^ ILC2 and Lin^-^Thy1.2^+^GATA3^+^IL-13^+^ ILC2 were enhanced when compared with WT mice **(** **Figure 4**).

Collectively, these results indicate that the constitutive loss of CD11c^hi^ cDCs enhances the AD-associated generation of ILC2, while it inhibits the emergence of T_eff_ cells and ILC3.

**Figure 5:**
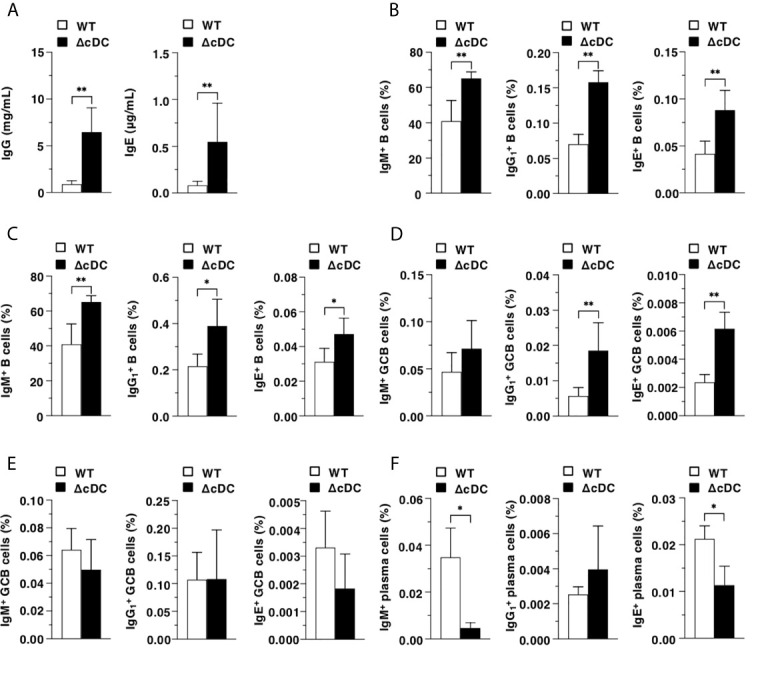
Constitutive loss of cDCs affects the AD-associated B-cell responses **in** during onset of AD-like inflammation. **(A)** Serum production of IgG and IgE in WT mice and ΔCD11c^hi^ cDC mice (n = 8 per group) at 16 days after topical application with MC903. **(B–E)** The frequency of the subsets of IgM^+^ B cells, $\text{Ig}{\text{G}}_{1}^{+}$ B cells, and IgE^+^ B cells in whole **(B, C)** and GC **(D, E)** of Spl **(B, D)** and EDLNs **(C, E)** obtained from WT mice and ΔCD11c^hi^ cDC mice (n = 8 per group) at 16 days after topical application with MC903. **(F)** The frequency of the subsets of IgM^+^ plasma cells, $\text{Ig}{\text{G}}_{1}^{+}$ plasma cells, and IgE^+^ plasma cells of BM obtained from WT mice and ΔCD11c^hi^ cDC mice (n = 8 per group) at 16 days after topical application with MC903. Data are obtained from eight individual samples in a single experiment. *P < .05, **P < .01 compared with WT mice by two-sided paired student *t*-test. All data are representative of at least 3 independent experiments.

We further determined the influence of the constitutive ablation of CD11c^hi^ cDCs in B-cell responses during onset of AD-like inflammation. ΔCD11c^hi^ cDC mice retained higher serum productions of IgG and IgE than WT mice (**Figure 5A**). Furthermore, ΔCD11c^hi^ cDC mice showed higher proportions of IgM^+^ B cells, $\text{Ig}{\text{G}}_{1}^{+}$ B cells and IgE^+^ B cells in Spl and EDLNs (**Figures 5B, C**). In addition, the frequencies of $\text{Ig}{\text{G}}_{1}^{+}$ B cells and IgE^+^ B cells in GC of Spl, but not that in EDLNs, were enhanced in ΔCD11c^hi^ cDC mice when compared with WT mice (**Figures 5D, E**). On the other hand, ΔCD11c^hi^ cDC mice exhibited lower proportion of IgM^+^ plasma cells and IgE^+^ plasma cells in BM than WT mice (**Figure 5F**).

Taken together, these results indicate that the constitutive deficiency of CD11c^hi^ cDCs strengthens B-cell responses for the reinforced secretion of Ab during progression of AD-like inflammation.

### Constitutive Loss of cDCs Promotes the Colonization of *S. aureus* in Eczematous Skin

**Figure 6:**
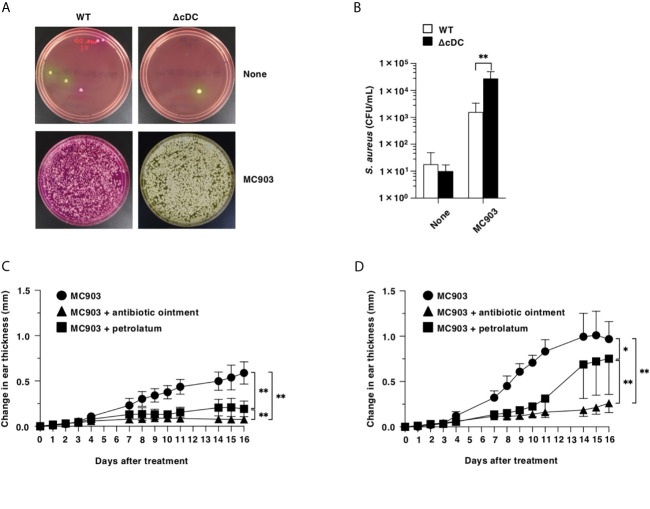
Constitutive loss of cDCs accelerates the colonization of *S. aureus* and the disruption of barrier function in eczematous skin. **(A, B)** Quantification of *S. aureus* cultured from the homogenates of ear skin of WT mice and ΔCD11c^hi^ cDC mice (n = 7 per group) at days 0 and 16 after topical application with MC903. **(A)** Representative pictures of Mannitol salt agar with egg yolk plates at days 0 (None) and 16 (MC903). **(B)** CFU of *S. aureus* at days 0 (None) and 16 (MC903) after topical application with MC903. **P <.01 compared with WT mice by two-sided paired student *t*-test. **(C, D)** WT mice **(C)** and ΔCD11c^hi^ cDC mice **(D)** (n = 6 per group) were treated topically with MC903 in combination with or without antibiotic ointment or petrolatum on the left ear skin. Ear thickness was evaluated for 16 days. Data are obtained from seven individual samples in a single experiment. *P < .05, **P < .01 compared with MC903-treated group or among groups. All data are representative of at least 3 independent experiments by two-way ANOVA.

To address the differences in the colonization of *S. aureus* in eczematous lesion between WT mice and ΔCD11c^hi^ cDC mice after topical treatment of MC903, we cultured the homogenates of their ear skin on Mannitol salt agar (16) after topical application of MC903, and quantified colony-forming units (CFU) of *S. aureus*. While of *S. aureus* was barely detected in the ear skin surface of WT mice and ΔCD11c^hi^ cDC mice in the homeostatic conditions, ΔCD11c^hi^ cDC mice showed a significant outgrowth of *S. aureus* in the eczematous lesions as compared with WT mice upon topical treatment of MC903 **(** **Figures 6A, B**).

We also examined the association of a dramatic increased colonization of *S. aureus* with the exacerbated AD-like inflammation under the constitutive lack of CD11c^hi^ cDCs **(** **Figures 6C, D**). Similarly observed in WT mice, the application of antibiotic ointment to ear skin after topical application of MC903 markedly attenuated the development of AD-like inflammation in ΔCD11c^hi^ cDC mice.

To investigate the role of the compromised skin barrier function for the enhanced eczematous inflammation under the constitutive deficiency of CD11c^hi^ cDCs, we coated the ear skin of ΔCD11c^hi^ cDC mice with petrolatum following topical application of MC903 (**Figures 6C, D**). Treatment of ΔCD11c^hi^ cDC mice with petrolatum prevented from forming eczematous lesions, while this treatment had a more prominent protecting effect in WT mice.

Taken together, these results indicate that the constitutive deficiency of CD11c^hi^ cDCs accelerates the colonization of *S. aureus* and the disruption of barrier function in eczematous skin for developing AD-like inflammation.

## Discussion

While the lineage of DCs are believed to be crucial for the initiation and development of AD in humans and rodent models of AD-like inflammation, their intrinsic role for the onset of the eczematous inflammation remains to be determined. In this study, our findings reveal that CD11c^hi^ cDCs are dispensable for the development of AD-like inflammation, while the homeostatic feedback loop between CD11c^hi^ cDCs and other leukocytes prevents the progression of AD-like inflammation. Thus, CD11c^hi^ cDCs could maintain skin immune homeostasis in the steady-state conditions to protect from the exacerbating the onset of eczematous inflammation.

Analysis of ΔCD11c^hi^ cDC mice showed that the constitutive absence of CD11c^hi^ cDCs led to the abnormal composition of leukocytes in lymphoid tissues, that associated with the reduced or enhanced proportions of T cell-subsets or granulocytes under steady-state conditions. On the other hand, ΔCD11c^hi^ cDC mice exhibited an increased serum amounts of Flt3L in the homeostatic conditions. Since Flt3 is expressed on shared progenitors of lymphocytes and all myeloid cells during the early stages of hematopoiesis, Flt3L is critical for the expansion of certain hematopoietic progenitors and the generation of several mature leukocytes (40–42). Furthermore, it has been shown that Flt3^+^ common myeloid progenitors (CMPs) and their downstream Flt3^+^ progenitors gave rise to cDCs, which were only mature peripheral leukocytes expressing Flt3 (40, 43). Giving the importance of Flt3L for the development and homeostatic maintenance of all stages of cDC lineage, they might be responsible for major consumption of Flt3L *in vivo*. These phenomena led us to hypothesize that the absence of CD11c^hi^ cDCs causes the release of the excess amounts of Flt3L in periphery. Although this possibility remains elusive, the elevated level of Flt3L in the constitutive absence of CD11c^hi^ cDCs would mainly promotes the differentiation of granulocytes as well as other cell types, resulting in the abnormal composition of leukocytes in the steady-states.

We showed that the constitutive absence of CD11c^hi^ cDCs elevated the productions of cytokines related to type 2/type 17 immune responses in periphery and cutaneous tissues, linking to the spontaneous systemic and cutaneous inflammatory responses regardless of the homeostatic conditions. Furthermore, the constitutive loss of CD11c^hi^ cDCs enhanced or inhibited the expression levels of alarmin or the barrier-related molecules, that associated with cutaneous inflammation and barrier dysfunction (44, 45). Thus, these phenomena imply that the abnormal immune constitution might reduce their threshold of responsiveness to sense endogenous damage-associated molecular patterns (DAMPs) to trigger the inflammation in periphery and cutaneous tissues, and that affects epidermal functionality.

Given the impairment of Ag-specific priming of T cells and reduction in the generation of T_H_1 cells and T_H_2 cells in lymphoid tissues in the constitutive absence of CD11c^hi^ cDCs under steady-state conditions, CD11c^hi^ cDCs could be prerequisite for induction of Ag-specific T_eff_-responses. On the other hand, the constitutive deletion of CD11c^hi^ cDCs enhanced the proportions of ILC2 and ILC3 in lymphoid tissues in the homeostatic conditions. Having demonstrated the critical role of Flt3L for the homeostatic maintenance of ILC2 and ILC3 by acting on early ILC progenitors (42, 46), the increased serum amounts of Flt3L in the absence of CD11c^hi^ cDCs could accelerate the development of ILC2 and ILC3, leading to the promotion of type 2/type 17 immune responses in periphery and cutaneous tissues.

We showed that the constitutive ablation of CD11c^hi^ cDCs not only elevated serum productions of IgG and IgE but also enhanced the generation of IgM^+^ B cells, $\text{Ig}{\text{G}}_{1}^{+}$ B cells, and IgE^+^ B cells in lymphoid tissues. It has been reported that Flt3 is expressed on pre-pro B cells and pre B cells, and Flt3L promotes the survival of Flt3^+^CD19^-^ progenitors (41, 42), supporting a critical role for Flt3L in early B-cell development. On the other hand, it has been shown that ILC2 and ILC3 enhances B-cell proliferation and Ab production in T-cell independent manner (47–50). Therefore, the massive production of Flt3L under the deficiency of CD11c^hi^ cDCs could promote the generation of ILC2/ILC3 that stimulates B-cell responses. Taken together, our findings suggest that Flt3L mediates the homeostatic feedback loop in the context of CD11c^hi^ cDCs/ILC2/ILC3/B cells under the steady-state conditions.

Different from the implications based on the previous findings with the critical role of CD11c^hi^ cDCs for the induction of T_H_2-responses during the development of allergic cutaneous inflammation (19–29), we revealed the aggressive development of the MC903-induced AD-like inflammation under the constitutive absence of CD11c^hi^ cDCs, accompanied by the abnormality in the composition of leukocytes and the enforced peripheral and cutaneous inflammatory status as well as the barrier dysfunction in eczematous lesions. While the constitutive loss of CD11c^hi^ cDCs reduced the generation of T_H_1 cells and T_H_2 cells as well as ILC1 and ILC3 in lymphoid tissues during the development of MC903-induced AD-like inflammation, it not only promoted the generation of ILC2 in lymphoid tissues but also enhanced serum productions of IgG and IgE, linking with increased emergence of IgM^+^ B cells, $\text{Ig}{\text{G}}_{1}^{+}$ B cells, and IgE^+^ B cells in lymphoid tissues and decreased generation of IgM^+^ plasma cells and IgE^+^ plasma cells. Given the sustained massive serum production of Flt3L under the constitutive deficiency of CD11c^hi^ cDCs, the breakdown of the Flt3L-mediated homeostatic feedback loop among CD11c^hi^ cDCs/granulocytes/ILC2/B cells could be responsible for the exacerbation of the eczematous inflammation.

The constitutive loss of CD11c^hi^ cDCs promoted the colonization of *S. aureus* in the eczematous ear skin, whereas the application of antibiotic ointment suppressed the progression of the MC903-induced AD-like inflammation. On the other hand, the treatment of the compromised skin barrier function with petrolatum mitigated the severity of the MC903-induced AD-like inflammation under the constitutive absence of CD11c^hi^ cDCs. Given the critical role of filaggrin in the formation of the functional skin barrier, the reduced expression of filaggrin caused the elevated skin pH during the development of AD, and that facilitates the colonization of *S. aureus* in the eczematous lesions, leading to the promotion of the eczematous inflammation (51–55). Therefore, the immune abnormality under the constitutive loss of CD11c^hi^ cDCs could enforce the eczematous inflammation in the skin accompanied by the reduced expression of filaggrin, and the disturbed skin barrier function could drive to the abundant colonization of *S. aureus* for exacerbation of the MC903-induced AD-like inflammation. Taken together, our findings suggest that the existence of CD11c^hi^ cDCs maintain the immune homeostasis, linking to the epidermal barrier function to prevent the massive colonization of *S. aureus* for protecting from AD flare.

In conclusion, our results unravel the unexpected role of CD11c^hi^ cDCs in the initiation and progression of AD-like inflammation. It has been highly appreciated that CD11c^hi^ cDCs are required for Ag-specific priming of CD4^+^ T cells to differentiate pathogenic T_H_2 cells for the development of AD (19–29). However, CD11c^hi^ cDCs could provide the Flt3L-mediated homeostatic feedback loop in the context of type 2 immune responses composed of granulocytes/ILC2/B cells, and that is critical link to skin barrier functions to limit the abundant colonization of *S. aureus* for protecting AD flare. Taken together, our findings propose that CD11c^hi^ cDCs maintain immune homeostasis to prevent the occurrence of the immune abnormalities skewing to type 2 immune responses for inhibiting the onset of eczematous inflammation. Thus, CD11c^hi^ cDCs and their feedback control may constitute the attractive targets for the intervention and treatment of allergic cutaneous disorders.

## Data Availability Statement

The original contributions presented in the study are included in the article/**Supplementary Material**. Further inquiries can be directed to the corresponding author.

## Ethics Statement

The animal study was reviewed and approved by The Animal Experiment Committee and Gene Recombination Experiment Committee at the University of Miyazaki.

## Author Contributions

KS designed all experiments, analyzed data and wrote the manuscript. YN, ToF, TaF, TU, HT, JN, NM, and NC did experiments. DR, YH, and MA provided reagents and information. All authors contributed to the article and approved the submitted version.

## Funding

This work was supported by a Grant-in-Aid for Scientific Research (B) (KS; 18H02670), for challenging Exploratory Research (KS; 16K15291), and for Young Scientists (B) (TU; 17K15027, ToF; 17K15732, and HT; 18K15194) from the Ministry of Education, Science and Culture of Japan, the Project for Cancer Research And Therapeutic Evolution (P-CREATE) from Japan Agency for Medical Research and development (AMED) (KS; 16cm0106307h0001, 17cm0106307h0002, 18cm0106307h0003, and 19cm0106307h0004), the Uehara Memorial Foundation (HT), Takeda Science Foundation (TU and ToF), the Naito Foundation (KS), Bristol-Myers Squibb Foundation Grants (KS), GSK Japan Research Grant 2016 (ToF), GSK Japan Research Grant 2017 (HT), GSK Japan Research Grant 2018 (TU), Daiichi Sankyo Foundation of Life Science (KS), Nipponham Foundation for the Future of Food (HT), and the Shin-Nihon Foundation of Advanced Medical Research (TU), and Nazarbayev University Faculty Development Grant (DR; 090118FD5310). GSK Japan was not involved in the study design, collection, analysis, interpretation of data, the writing of this article or the decision to submit it for publication.

## Conflict of Interest

The authors declare that the research was conducted in the absence of any commercial or financial relationships that could be construed as a potential conflict of interest.

## Abbreviations

Ab, Antibody; AD, Atopic dermatitis; Ag, Antigen; APCs, Ag-presenting cells; BM, Bone marrow; cDCs, Conventional dendritic cells; CFU, Colony-forming units; CMPs, Common myeloid progenitors; DAMPs, Damage-associated molecular patterns; DT, Diphtheria toxin; DTA, DT α chain; EDLNs, Ear-draining lymph nodes; ELISA, Enzyme-linked immunosorbent assay; Flt3L, Fms-related tyrosine kinase 3 ligand; GC, Germinal center; HPF, High performance field, H&E, Hematoxylin and eosin, IFN, Interferon; Ig, Immunoglobulin; IL, Interleukin; ILCs, Innate lymphoid cells; i.p., Intraperitoneal; i.v., Intravenously; mAb, Monoclonal Ab; MCs, Mast cells; MHC, Major histocompatibility complex; NKT, Natural killer T; OVA, Ovalbumin; pDCs, Plasmacytoid DCs; PFA, Paraformaldehyde; RT-PCR, Reverse transcription polymerase chain reaction; Spl, Spleen; *S. aureus*, *Staphylococcus aureus*; TCR, T-cell receptor; T_eff_, Effector T; Tg, Transgenic; T_H_2 cells, Type 2 helper T; TLR, Toll-like receptor; TNF, Tumor necrosis factor; T_reg_, Regulatory T; WT, Wild-type.
