# Supplementary material for: Congenital Deficiency of Conventional Dendritic Cells Promotes the Development of Atopic Dermatitis-Like Inflammation
Source: Front Immunol. 2021 Jul 27;12:712676. doi: 10.3389/fimmu.2021.712676 (PMC8356667; doi:10.3389/fimmu.2021.712676)
Supplement: Supplementary file 1 [file DataSheet_1.pdf]

Supplementary Material

Congenital Deficiency of Conventional Dendritic Cells Promotes the Development of Atopic Dermatitis-Like Inflammation

Yotaro Nishikawa, Tomohiro Fukaya, Takehito Fukui, Tomofumi Uto, Hideaki Takagi, Junta Nasu, Noriaki Miyanaga, Dieter Riethmacher, Narantsog Choijookhuu, Yoshitaka Hishikawa, Masahiro Amano and Katsuaki Sato*

*** Correspondence:** Katsuaki Sato: katsuaki_sato@med.miyazaki-u.ac.jp

SUPPLEMENTARY MATERIALS AND METHODS

Tissue and Cell Isolation

Leukocytes were prepared from Spl, EDLNs, and BM as described previously (4, 5, 9, 33-37). In brief, Spl and EDLNs were digested with 400 U/ml collagenase type III (Worthington Biochemical) at 37 ºC for 20 to 30 min, and were ground between glass slides. BM cells were flushed from the femurs and tibias. Cell suspensions of Spl and BM were further treated with RBC lysis buffer (Sigma-Aldrich). Ear skin tissue was obtained by separating the dermal sheets of the ear and digesting with 400 U/ml collagenase type III at 37 ºC for 60 min and cut into pieces. Epidermis was obtained by separating the dermal sheets of the ear and digesting in 0.1% Trypsin-EDTA (Gibco) and 10 mg/ml DNAse (Roche) at 37 ºC for 50 min. Single-cell suspensions were obtained by forcing through a 40- or 100-μm cell strainer (BD Biosciences). $CD4^+$ T cells or $CD8^+$ T cells were purified from splenocytes of B6.CD45.1$^+$OT-II mice (CD45.1$^+$Vα2$^+$OT-II $CD4^+$ T cells) or B6.CD45.1$^+$OT-I mice (CD45.1$^+$Vα2$^+$OT-I $CD8^+$ T cells) with mouse CD4 T lymphocyte Enrichment Set-DM and (BD Biosciences) or mouse CD8 T lymphocyte Enrichment Set-DM (BD Biosciences).

Flow Cytometry

Cells were stained with fluorescein-conjugated monoclonal Abs (mAbs) listed in **TABLE S1**. For the intracellular expression of cytokines (4, 5, 9, 33-37), cells were incubated for 4 hrs with phorbol 12-myristate 13-acetate (PMA, 50 ng/ml; Sigma-Aldrich) and ionomycin (IoM, 500 ng/ml; Sigma-Aldrich) plus GolgiPlug (BD Biosciences) during the final 2 hrs. Subsequently, the cells were resuspended in Fixation-Permeabilization solution (eBiosciences) and intracellular cytokine staining was carried out according to the manufacturer's directions. Fluorescence staining was analyzed with a FACSVerse flow cytometer (BD Biosciences) and FlowJo software (Tree star).

Quantitative Reverse Transcription Polymerase Chain Reaction (RT-PCR)

Total RNA from cells was extracted by using ISOGEN II (Nippon Gene) and the first-strand complementary DNA (cDNA) was synthesized from 100 ng of total RNA with oligo$(dT)_{20}$ primer using the PrimeScript RT Master Mix (Takara) according to the manufacturer's instructions. Transcriptional expression levels were analyzed as described previously (9, 36) by using SYBR® Premix Ex Taq II on Thermal Cycler Dice (Takara) with specific primer pairs listed in **TABLE S1** after normalization for *bactin* expression.

Measurement of Serum Ig, Fms-Related Tyrosine Kinase 3 Ligand (Flt3L), and Cytokines

Serum total IgG and total IgE were assayed by using Mouse IgG enzyme-linked immunosorbent assay (ELISA) kit (Bethyl Laboratories) and Mouse IgE ELISA MAX kit (BioLegend). Serum Flt3L was measured by using Mouse/Rat Flt-3L Ligand/FLT3L Quantikine ELISA kit (R&D Systems). Serum TNF-α, IFN-γ, IL-2, IL-4, IL-5, IL-6, IL-10, IL-13, IL-17A, and IL-22 were analyzed by flow cytometry using LEGENDplex™ MU Th Cytokine Panel (13-plex) (BioLegend). All experiments were performed according to the manufacturer's instructions.

AD-Like Inflammation

To induce AD-like skin lesions (13, 15, 38-40), mice received topical application of 2 nmol of MC903 (calcipotriol, Sigma-Aldrich) in 20 μl of ethanol (vehicle) on the left ear skin for five consecutive days a week during 2 weeks followed by two consecutive days a week during last week for 16 days. As vehicle control, the same amount of ethanol was applied. The development of AD-like inflammation was determined by ear thickness at every day after topical application with MC903 for 16 days using digital calipers (PK-1012CPX; Mitsutoyo) (9, 37). In some experiments, Spl, EDLNs, and ear were obtained from the mice before or after the first topical application of MC903 at 16 days. For the improvement of the compromised skin barrier function, petrolatum (Wako Pure Chemicals) was topically applied twice daily at the days of MC903 application to the ventral and dorsal portions of the ear for the duration of the treatment with MC903. For antibiotic treatment, mice were treated topically with 1% Acuatim ointment (Nadifloxacin, Otsuka) on the ventral and dorsal portions of the ear twice daily at the days of MC903 application for the duration of the treatment with MC903.

Adoptive Transfer

For Ag-specific priming of $CD4^+$ T cells or $CD8^+$ T cells in vivo(4,33-36), $CD45.1^+$OT-II $CD4^+$ T cells or $CD45.1^+$OT-I $CD8^+$ T cells were labeled with eFluor™ 670 (Thermo Fisher Scientific ; 2.5 μM) at 37 °C for 10 min, and washed twice with cold PBS. Subsequently, eFluor™ 670-labeled $CD45.1^+$OT-II $CD4^+$ T cells or $CD45.1^+$OT-I $CD8^+$ T cells (each 5×10^6/mouse) were intravenously (i.v.) injected into mice 24 hrs before intraperitoneal (i.p.) injection with OVA protein (A5503, Sigma-Aldrich). After 3 days, the gated $CD45.1^+$OT-II $CD4^+$ T cells or $CD45.1^+$OT-I $CD8^+$ T cells

in Spl and EDLNs were analyzed for eFluor™ 670 dilution to detect the dividing cells by flow cytometry.

Histopathologic Assessment

Tissues from the ear were fixed with 4% paraformaldehyde (PFA) in PBS and embedded in paraffin. The tissue sections (5 μm thickness) were stained with H&E and toluidine blue to detect MCs. The stained slides were examined with a bright-field microscopy (BX53; Olympus). The areas of the epidermis and dermis of ear were quantified by thickness using ImageJ (National Institutes of Health) by a blinded observer as described previously (9, 37).

Immunohistochemical Analysis

Spl and ear skin were embedded in OCT compound (Sakura Fineteck) and frozen in liquid N_2. Frozen section (5 μm) was fixed with cold acetone, and blocked with PBS containing 5% of normal rat serum and Avidin/Biotin Blocking Kit (Vector laboratories). Subsequently, slide of Spl was stained with Alexa Fluor 488-conjugated anti-B220/CD45R mAb, phycoerythrin (PE)-eFluor610-conjugated anti-CD11c mAb (eBiosciences), allophycocyanin (APC)-conjugated anti-CD3ε mAb (BioLegend), and mounted with ProLong Diamond (Thermo Fisher Scientific). Alternatively, slide of skin was stained with PE-conjugated anti-CD207 mAb (BioLegend) followed by horseradish peroxidase (HRP)-labeled goat anti-rat IgG. HRP-sites were visualized by fluorescein isothiocyanate (FITC)-tyramide conjugates and counterstained with DAPI (Invitrogen). The stained slides were analyzed with BZ-X710 fluorescence microscope (KEYENCE, Osaka, Japan).

Bacterial Culture

To measure CFU of *S. aureus* (16), the homogenates of ear skin were placed into 1 ml of PBS, and serial dilutions were prepared. 100 μl of each dilution was transferred on Mannitol salt agar with egg yolk plates (BD), and colonies were counted after 2-day incubation. CFUs were calculated by dividing the number of colonies per plate by the dilution factor.

TABLE S1. mAbs for flow cytometry

Name	Supplier	clone	conjugate
CD3ε	BD	145-2C11	BV421,BV510
CD3ε	Biolegend	145-2C11	PerCP-Cy5.5,APC,PE-Cy7
CD4	BD	RM4-5	PE,PE-Cy7
CD8α	BD	53-6.7	FITC,PE,APC
CD8α	Biolegend	53-6.7	APC-Cy7
CD11b	BD	M1/70	PE-Cy7,BV510
CD11b	Biolegend	M1/70	FITC
CD11c	BD	HL3	FITC,APC,BV421
CD19	BD	1D3	FITC
CD19	Biolegend	6D5	BV510
CD44	BD	IM7	BV510
CD45	Biolegend	30-F11	FITC,APC-Cy7
CD45R/B220	BD	RA3-6B2	Alexa-488
CD45R/B220	Biolegend	RA3-6B2	PE-Cy7,APC-Cy7
CD45.1	BD	A20	FITC
CD45.2	Biolegend	104	APC-Cy7
CD45.2	Tonbo Biosciences	104	violetFluor 450
CD49b	BD	DX5	FITC,APC
CD90.2	Biolegend	6D5	BV510
CD103	BD	M290	APC
CD117	BD	2B8	PE-Cy7
CD127	Biolegend	A7R34	PE
CD138	Miltenyi	REA104	PE
CD207	Biolegend	4C7	PE
CD335	Biolegend	29A1.4	PE-Cy7
CXCR5	Biolegend	L138D7	APC
Fas	BD	Jo2	PE
FcεRIα	eBioscience	MAR-1	PE,APC
Foxp3	eBioscience	FJK-16s	APC
Gata-3	eBioscience	TWAJ	PerCP-eFluor710
GL7	Biolegend	GL7	Bacific Blue
Gr-1	Biolegend	RB6-8C5	FITC
IFN-γ	eBioscience	XMG1.2	APC
IgE	Biolegend	RME-1	FITC
IgG1	BD	A85-1	APC
IgM	Biolegend	RMM-1	APC
IL-4	Biolegend	11B11	Alexa-488
IL-5	Biolegend	TRFK5	BV421
IL-13	eBioscience	eBio13A	PE
IL-17A	BD	TC11-18H10	PE
I-A/I-E	Biolegend	M5/114.15.2	APC-Cy7,BV510
Ly6G	Biolegend	1A8	FITC
NK1.1	BD	PK136	FITC
PD-1	BD	J43	PE
RORγt	BD	Q31-378	BV421
Siglec-F	Miltenyi	REA798	PE
Siglec-H	Biolegend	551	PE
ST2	BD	U29-93	BV421
T-bet	Biolegend	4B10	PE
TCR-β	Biolegend	GL3	PE
TCR γ/δ	Biolegend	GL3	APC,BV421
TER-119	Biolegend	TER-119	FITC

TABLE S2. Primers and sequences for RT-PCR

RT-PCR primer	Sequence
bactin	F-5'-ATCATTGCTCCTCCTGAGCG-3'<br>R-5'-GCTGATCCACATGTCCTGGAA-3'
Filaggrin	F-5'-GCTGAAGGAACTTCTGGAAAAG-3'<br>R-5'-GCCAACTTGAATACCATCAGAAG-3'
Il4	F-5'-GGCATTTTGAACGAGGTCAC-3'<br>R-5'-AAATATGCGAAGCACCTTGG-3'
Il5	F-5'-AGCACAGTGGTGAAAGAGACCTT-3'<br>R-5'-TCCAATGCATAGCTGGTGATTT-3'
Il13	F-5'-GGAGCTGAGCAACATCACACA-3'<br>R-5'-GGTCCTGTAGATGGCATTGCA-3'
Il17a	F-5'-CTGCTGAGCCTGGCGGCTAC-3'<br>R-5'-CATTGCGGTGGAGAGTCCAGGG-3'
Il22	F-5'-CAGCTCCTGTCACATCAGCGGT-3'<br>R-5'-AGGTCCAGTTCCCCAATCGCCT-3'
Il25	F-5'-ACAGGGACTTGAATCGGGTC-3'<br>R-5'-TGGTAAAGTGGGACGGAGTTG-3'
Il31	F-5'-ACAACTATAGCATAAAGCAGGC -3'<br>R-5'-GATTCATCAGTATTTCCAGGCA-3'
Il33	F-5'-GCTGCGTCTGTTGACACATT-3'<br>R-5'-CACCTGGTCTTGCTCTTGGT-3'
Loricrin	F-5'-CACATCAGCATCACCTCCTTCC-3'<br>R-5'-CCTCCTCCACCAGAGGTCTTTC-3'
Reg3g	F-5'-TTCCTGTCCTCCATGATCAAAA-3'<br>R-5'-CATCCACCTCTGTTGGGTTCA-3'
Saa1	F-5'-TGTTCACGAGGCTTTCCAAG-3'<br>R-5'-CCCGAGCATGGAAGTATTTG-3'
Saa2	F-5'-TGCAAGAGAGAGCTTTCAGG-3'<br>R-5'-TCAGTATTTGGCAGGCAGTC-3'
S100a7	F-5'-GGGCAGCTGACAAAAACAAG-3'<br>R-5'-TGGAACTGGAGATGGTAGTCC-3'
S100a8	F-5'-CCATGCCCTCTACAAGAATG-3'<br>R-5'-ATCACCATCGCAAGGAACTC-3'
S100a9	F-5'-CAGCATAACCACCATCATCG-3'<br>R-5'-AAGGTTGCCAACTGTGCTTC-3'
Tarc	F-5'-AGTGGAGTGTTCCAGGGATG-3'<br>R-5'-GTCACAGGCCGCTTTATGTT-3'
Tslp	F-5'-ACGGATGGGGCTAACTTACAA-3'<br>R-5'-AGTCCTCGATTTGCTCGAACT-3'

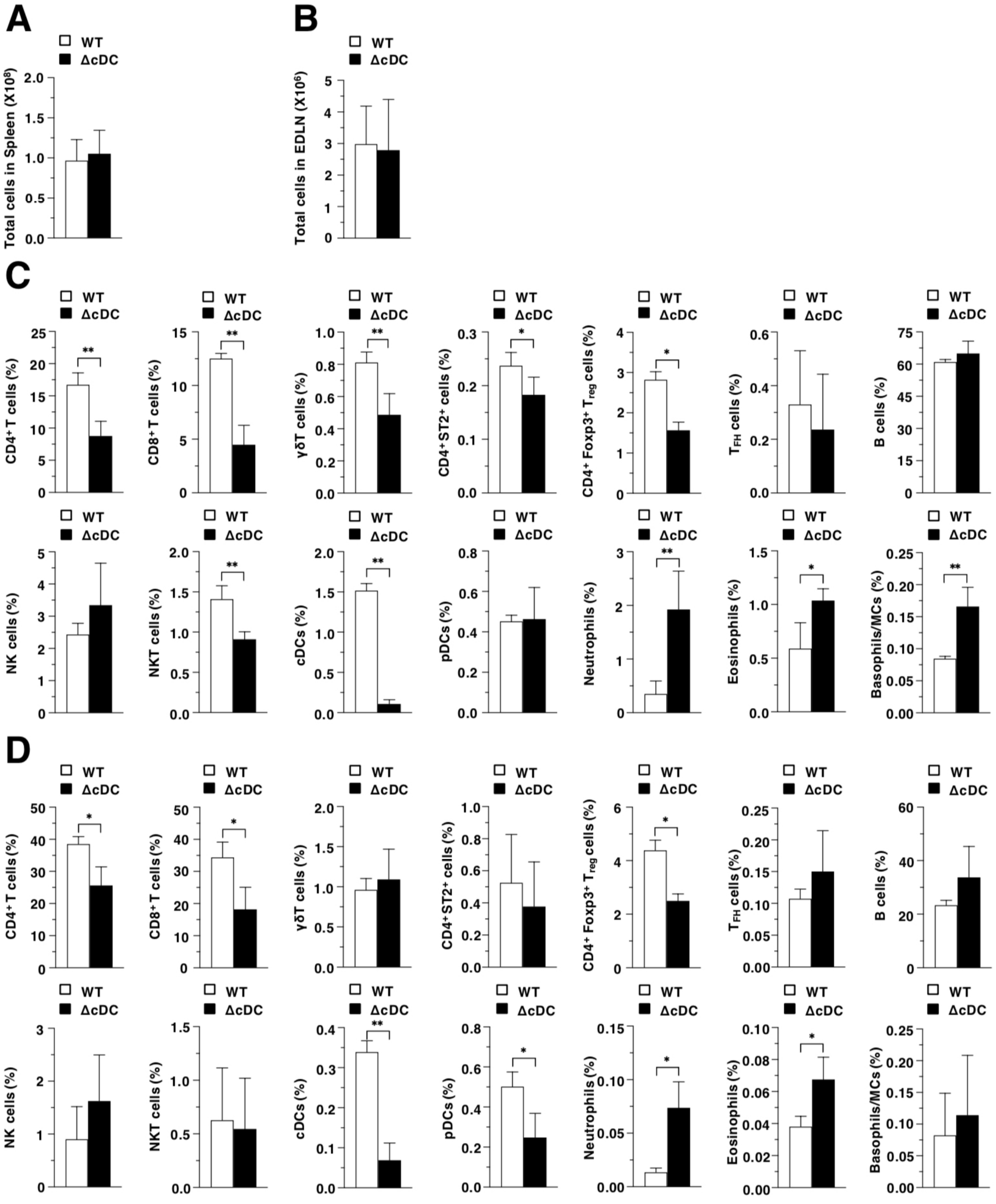

FIGURE S1. Constituencies of leukocytes in ΔCD11c^{hi} cDC mice in the homeostatic conditions. (**A,B**) Absolute cell numbers of leukocytes in Spl (**A**) and EDLNs (**B**) obtained from WT mice and ΔCD11c^{hi} cDC mice (n = 7 per group). (**C,D**) The frequency of leukocytes in Spl (**C**) and EDLNs (**D**) obtained from WT mice and ΔCD11c^{hi} cDC mice (n = 4 per group). Data are obtained from two to seven individual samples in a single experiment. $^{*}P < .05$, $^{**}P < .01$ compared with WT mice. All data are representative of at least 3 independent experiments.

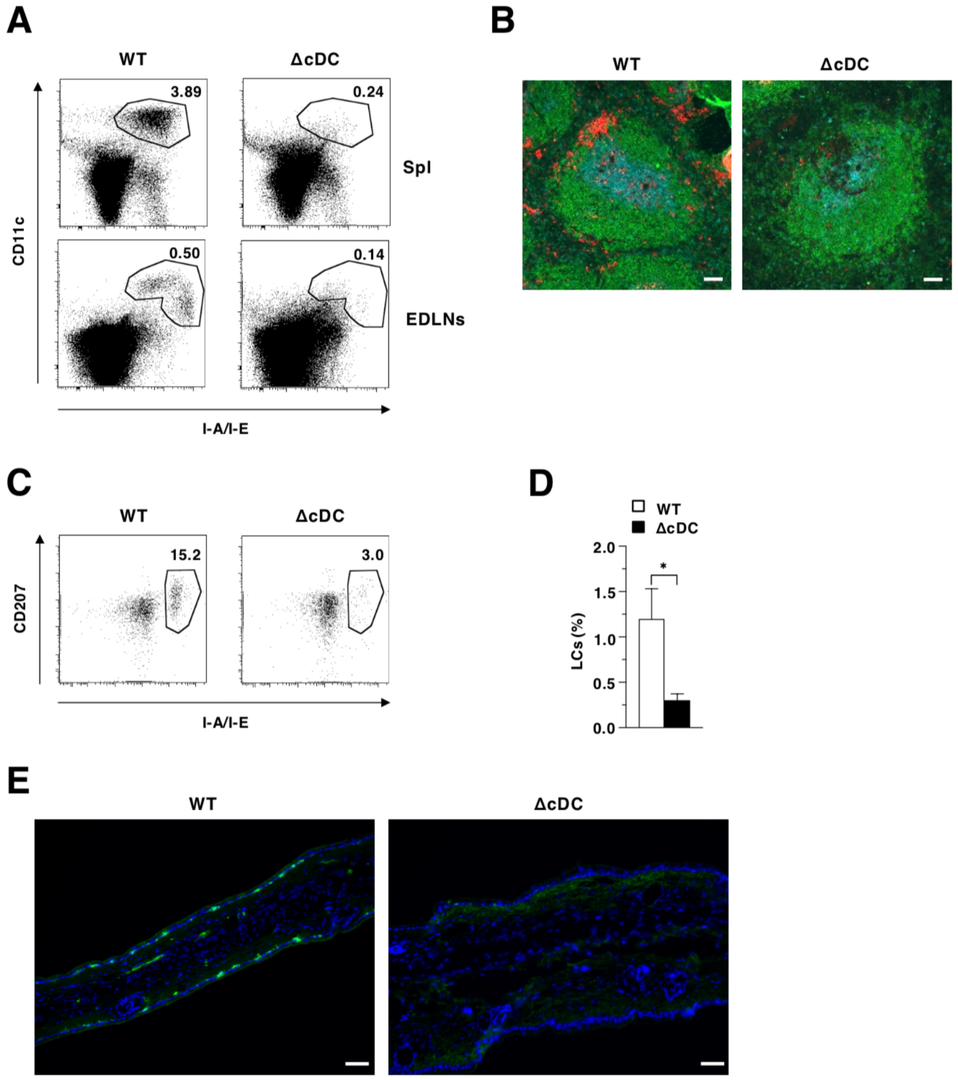

FIGURE S2. Lack of CD11c^{hi} cDCs in ΔCD11c^{hi} cDC mice under steady-state conditions. (**A**) Cell surface expression profile of CD11c^{+}I-A/I-E^{+} cDCs in Spl and EDLNs obtained from WT mice and ΔCD11c^{hi} cDC mice (n = 4 per group). (**B**) Frozen sections obtained from Spl obtained from WT mice and ΔCD11c^{hi} cDC mice (n = 2 per group) were stained for B220 (green), CD11c (red) and CD3ε (blue). Bars indicate 100 μm. (**C**,**D**) Cell surface expression profile (**C**) and proportion (**D**) of CD207^{+}I-A/I-E^{+} LCs in epidermis obtained from WT mice and ΔCD11c^{hi} cDC mice (n = 3 per group). (**E**) Frozen sections obtained from epidermis obtained from WT mice and ΔCD11c^{hi} cDC mice (n = 3 per group) were stained for CD207 (green) and DAPI (blue). Bars indicate 50 μm. Data are obtained from two to four individual samples in a single experiment. *$P < .05$ compared with WT mice. All data are representative of at least 3 independent experiments.

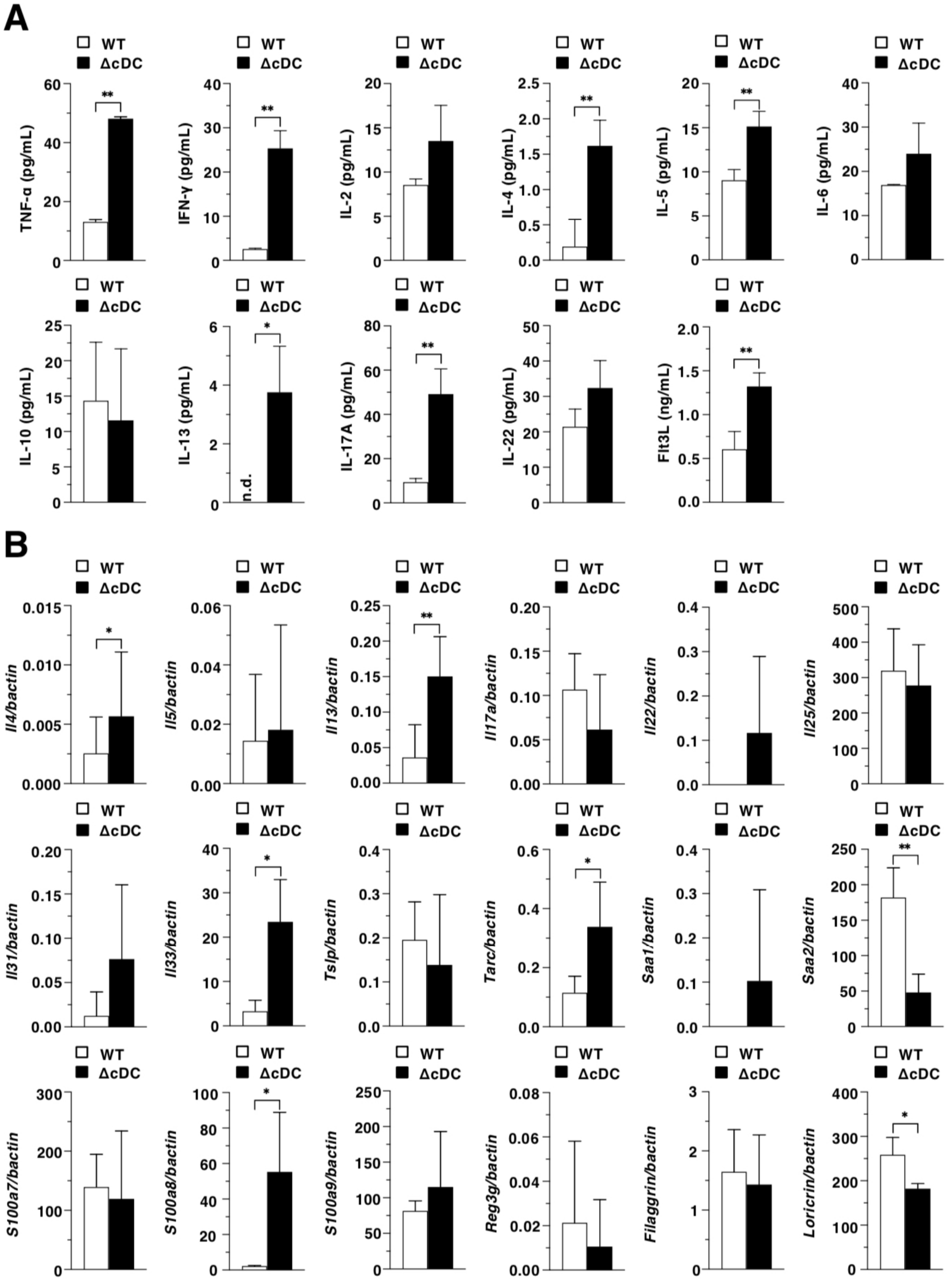

FIGURE S3. Inflammatory status of ΔCD11c^{hi} cDC mice in the homeostatic conditions. (**A**) Serum production of cytokines in WT mice and ΔCD11c^{hi} cDC mice (n =4 per group). n.d.; not determined. (**B**) Transcriptional expression of inflammation- and epithelium-related molecules in ear skin obtained from WT mice and ΔCD11c^{hi} cDC mice (n = 6 per group). Data are obtained from three to six individual samples in a single experiment. **P* < .05, ***P* < .01 compared with WT mice. All data are representative of at least 3 independent experiments.

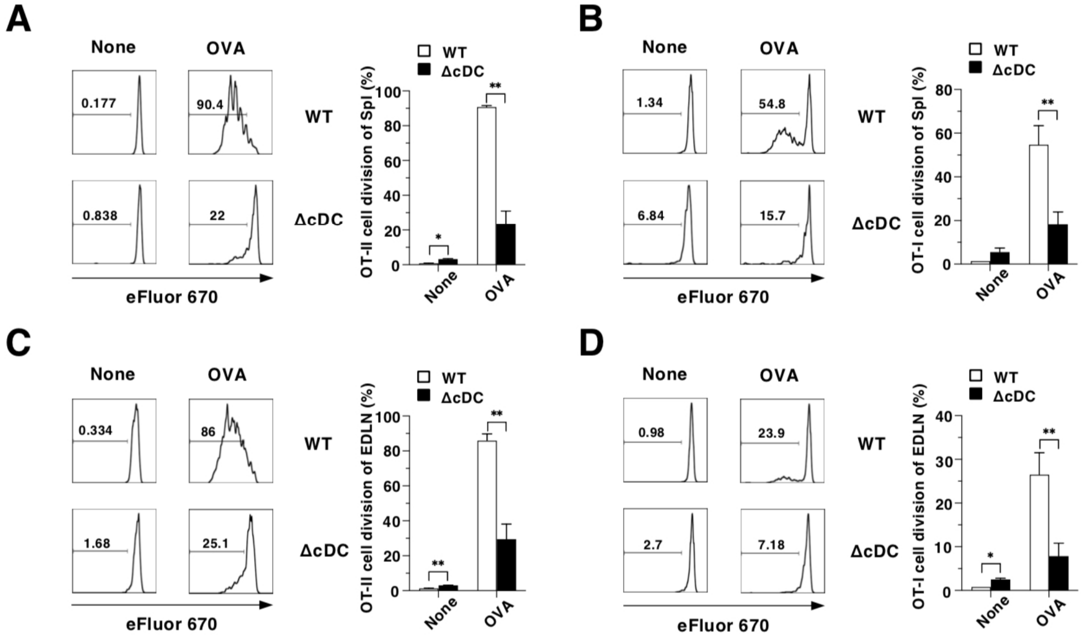

FIGURE S4. Ag-specific responses of T cells in $\Delta CD11c^{hi}$ cDC mice in the homeostatic conditions. WT mice and $\Delta CD11c^{hi}$ cDC mice (n = 3 per group) were adoptively transferred with eFluor™ 670-labelled $CD45.1^{+}$OT-II $CD4^{+}$ T cells (**A,C**) or $CD45.1^{+}$OT-I $CD8^{+}$ T cells (**B, D**), and then mice were systemically administered with OVA protein. Cell dividing profile (left panel) and proportion (right panel) of $CD45.1^{+}$OT-II $CD4^{+}$ T cells (**A,C**) or $CD45.1^{+}$OT-I $CD8^{+}$ T cells (**B,D**) in Spl (**A,B**) and EDLNs (**C,D**) at 3 days after the administration. Data are obtained from two to three individual samples in a single experiment. $^{**}P < .01$ compared with WT mice. All data are representative of at least 3 independent experiments.

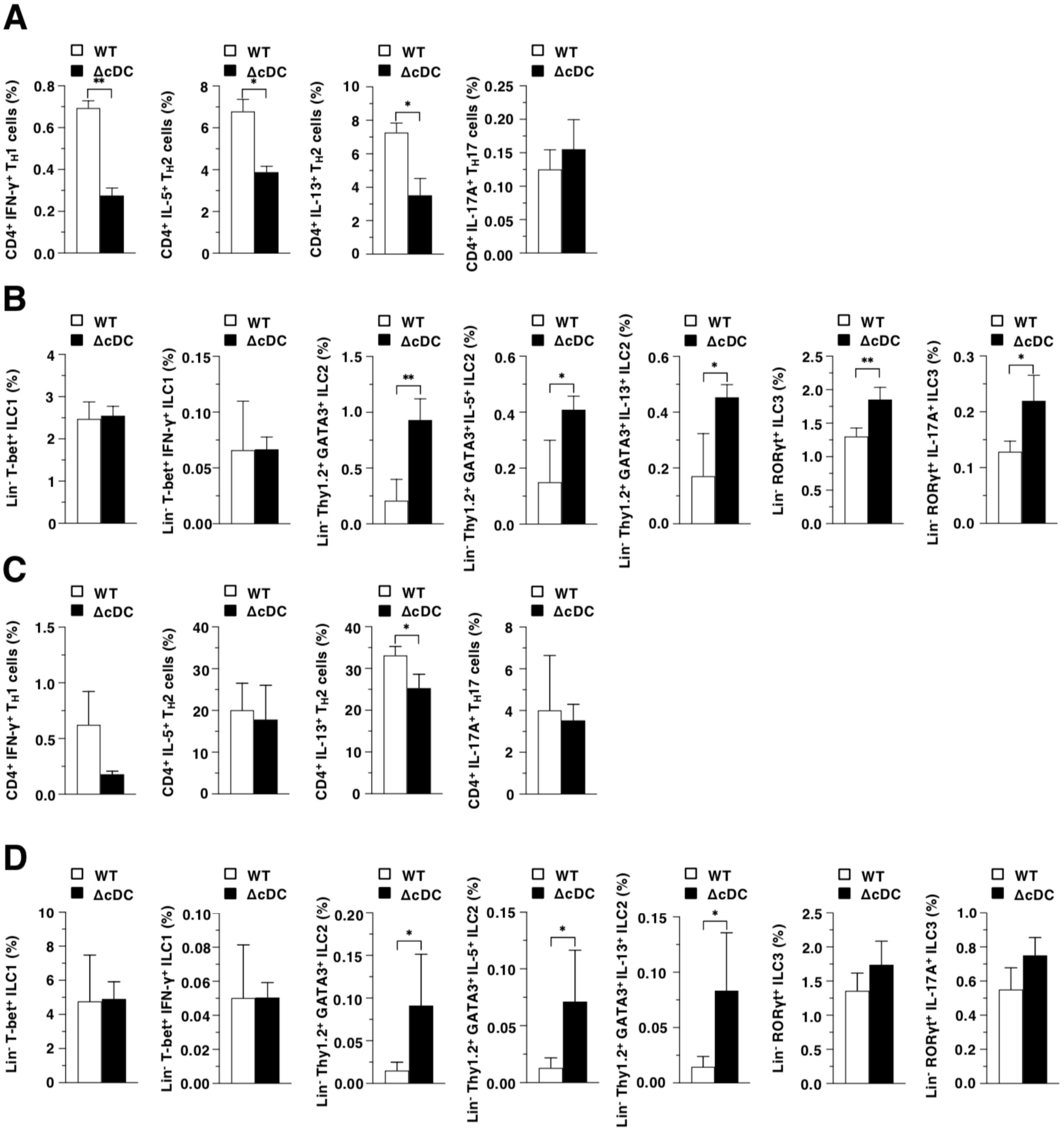

FIGURE S5. Composition of $CD4^+$ T_{eff} cells and ILCs in $\Delta CD11c^{hi}$ cDC mice in the homeostatic conditions. The frequency of the subsets of T cells (**A,C**) and ILCs (**B,D**) in Spl (**A,B**) and EDLNs (**C,D**) obtained from WT mice and $\Delta CD11c^{hi}$ cDC mice (n = 6 per group). Data are obtained from three to six individual samples in a single experiment. $*P < .05$, $**P < .01$ compared with WT mice. All data are representative of at least 3 independent experiments.

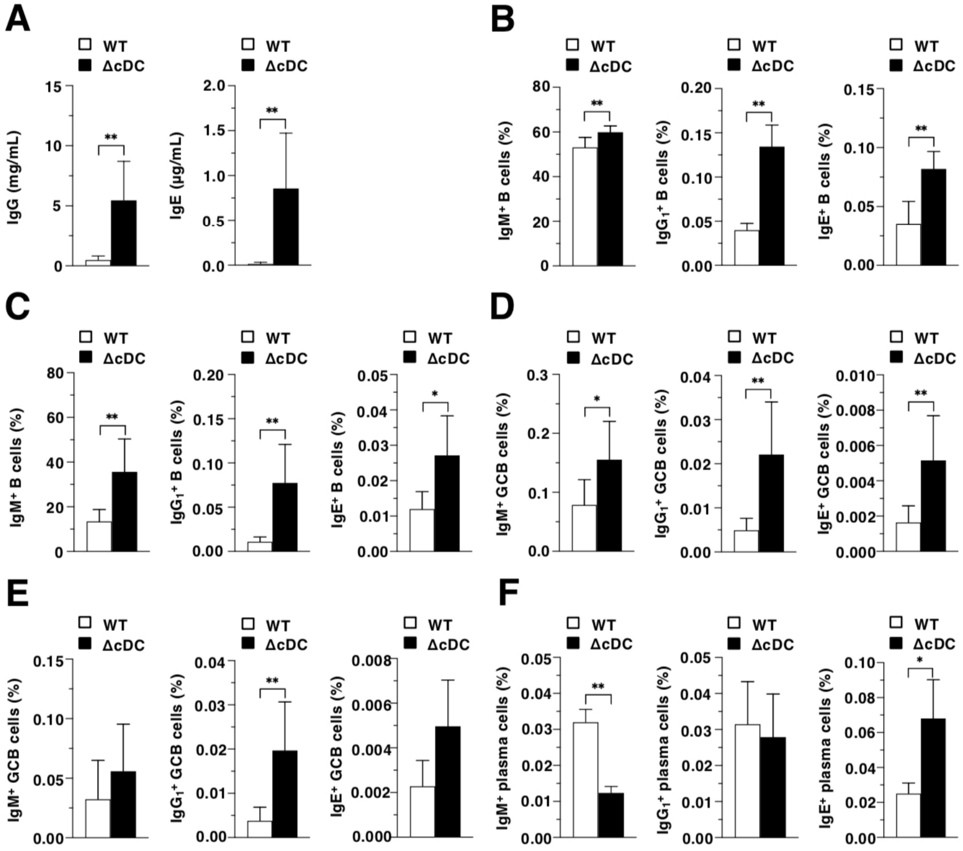

FIGURE S6. B-cell responses in ΔCD11c^{hi} cDC mice in the homeostatic conditions. (**A**) Serum production of IgG and IgE in WT mice and ΔCD11c^{hi} cDC mice. (**B-E**) The frequency of the subsets of IgM^{+} B cells, IgG$_1^{+}$ B cells, and IgE^{+} B cells in whole (**B,C**) and GC (**D,E**) of Spl (**B,D**) and EDLNs (**C,E**) obtained from WT mice and ΔCD11c^{hi} cDC mice (n = 8 per group). (**F**) The frequency of the subsets of IgM^{+} plasma cells, IgG$_1^{+}$ plasma cells, and IgE^{+} plasma cells of BM obtained from WT mice and ΔCD11c^{hi} cDC mice (n = 8 per group). Data are obtained from three to eight individual samples in a single experiment. **P* < .05, ***P* < .01 compared with WT mice. All data are representative of at least 3 independent experiments.
